# Notes and News

**Published:** 1890-07-05

**Authors:** 


### Notes and News.

Collected and Communicated.

The reviews are of particular interest to medical readers this month. In the *Nineteenth Century*, Dr. Herbert Snow gives it as his opinion that mental distress is the *vera causa* of cancer, which he declares to be largely on the increase. In the *Contemporary*, the Rev. B. Waugh follows up last month's article on baby-farming by one on "Child-Life Insurance." The subject of hydrophobia, or rather of M. Pasteur's treatment of this disease, is dealt with both in the *Contemporary* and the *National Review*. In the latter also is an article on "Charitable Endowments," which is worthy of attention.

Retford General Dispensary and Cottage Hospital seems to be more appreciated in its aspect as dispensary than as hospital. "In the dispensary department 1,032 patients' tickets have been received and the cases attended to. In the hospital department 27 cases have been treated, and the committee cannot but believe that, as its capabilities for treatment and the comfort it affords during illness become known, a far greater number of sufferers within reach (out of a population of fully 15,000) will avail themselves of them. The subscribers will notice the satisfactory state of the treasurer's accounts, showing a balance of £56 9s. 7½d. in hand." We quote from the report just issued. It is seldom that a hospital complains of too few patients.

Mr. W. H. Smith's "At Home" in aid of the Children's Country Holidays Fund was very successful, and brought together a fashionable company. The Chairman (Earl Fortescue) said that 20,772 children, taken from all parts of London, were last year sent into the country through the instrumentality of the fund. The selection was made by thirty-seven local committees, the members of which took a personal interest in the children. The expenditure of the fund last year was £13,178, towards which sum the payments of parents contributed £4,474. Thus the parents defrayed nearly one-third of the cost, which, including expenses of transit and organization, was about 13s. per head. Each child stayed in the country for not less than a fortnight.

The Lord Bishop of Cork presided at the annual meeting of friends of the County and City of Cork Hospital for Women and Children, in the beginning of the month. Some 402 in-patients were treated last year, and there were 11 deaths. The cost of maintenance per bed, excluding expenditure on alterations, etc., was £27 5s. 11d., being a highly satisfactory result. The receipts, excluding donations to Miss Baxter's special fund, amounted to £1,098 4s. 11d., and the expenditure, excluding payments under the same head, £1,313 9s. 3d., so that the income has not met the expenditure. The deficit would have been greater had not the Board received a bequest of £100 from the late Miss Cranitch. The Bishop and other speakers made a special appeal for funds, which it is hoped will meet with immediate response. The nursing staff and its excellent superintendent came in for much well-deserved praise.

We have been very gay lately: garden parties and soirées and afternoon teas have been frequent, but there is a strange melancholy over the social pleasures offered to the editor of a medical paper. It is not very exhilarating, if it is exciting, to go to a ball at a lunatic asylum, and even the garden party at the Cancer Hospital had an undercurrent of sadness. Then afternoon tea flavoured with anecdotes of cruelty to children, and followed by the handing round of a plate, is apt to grow tedious; and even a festival dinner, followed by speeches about incurables, is a rose with a thorn. We grumble not; all this and more we would willingly bear for the sake of those charities in whose cause we exist. But we do ask a little pity and a little patience from the inventors of patent medicines. Many of these are so very lowering to the system we really cannot afford to sample them during the very height of the season. We solemnly promise that all these bottles and pill boxes and patent foods, presented to us gratis, shall be attended to in turn—but grant us time. How nice it would be to be the editor of "The Gardener" this weather, and receive strawberries and green peas instead of pills and potions!

A death from hydrophobia has occurred at Stalybridge. On January 28th, an errand boy, aged 13, was walking along Huddersfield Road, when a large hound dog flew at him and bit him on the right hand. The animal was evidently suffering from rabies, and was pursued by two Stalybridge constables, whom it had previously attacked. The hound attacked a postman, who was bitten on the leg. It afterwards fiercely attacked a child six years old, whom it knocked down and commenced worrying, inflicting terrible wounds on the child's face and arms. The father of the child came out of his house to the rescue of his child, only to be bitten severely on one of his hands. A volunteer fetched his rifle and shot the brute. These three patients were immediately sent off to Paris to be treated by M. Pasteur. They have returned, and are now considered out of danger. In the case of the lad the wound in his hand did not bleed, and he attended to his employment until a little over two hours after he was bitten, when he told his step-mother what had happened. She took him to Dr. Chamberlayne, who ordered bread poultices, and they were applied. On the following morning he was seen by Mr. Hartley, of Colne, and Mr. Edwards, the chief constable of Clitheroe, and the boy underwent the Colne treatment. The wound gradually healed up, and the deceased did not make any complaint until the 19th instant, when he said he felt severe pain in his right arm, and the glands of his neck became swollen. Nothing was given him, however. On the 20th he had a pain in his chest, and goose-grease was applied. On Saturday he was no better, and Dr. Chamberlayne was again called in. On seeing the lad he ordered his removal to the infirmary, where he was at once taken. He died there from hydrophobia.

THE following vacancies are announced this week, the amount of the salary in each case being given after the name of the institution:—

**Resident Medical Officers.**

Wilts County Asylum, Assistant Medical Officer—£100, board, etc.

Stroud General Hospital, House Surgeon—£80, board, etc.

Ancoats Hospital, Manchester, House Surgeon—£80, board, etc.

Miller Hospital, Greenwich, Junior Medical Officer — £30, board, etc.

**Non-Resident Medical Officers.**

North-West London Hospital, Assistant Physician.
Gordon Hospital, Assistant Surgeon.
Queen's College, Birmingham, Lecturer on Public Health.

---

HERE is a message from far lands; the annual report of the Blackall District Hospital of Queensland, founded in 1880. Last year 149 in-patients and 96 out-patients were treated; the total income was £2,034, of which £1,287 is Government endowment. The hospital seems to be well and carefully managed, and the nursing is done by Mr. and Mrs. French.

---

THERE is one stream which never dries up in this bonnie England of ours, and that is the stream of charity. On Saturday last the chairman of the Norfolk and Norwich Hospital announced that Mr. B. E. Fletcher was going to build and furnish a convalescent home for them, and to endow it to the extent of £100 a-year; also that the Earl of Leicester had promised £15,000 towards the endowment fund. Quite lately the Dowager Lady Howard de Walden gave £1,200 to build a children's ward to Kilmarnock Infirmary. So long as the rich give their thousands of pounds, and our workmen give their pennies, we can proudly repel the proposals for "State aid" for our charities.

---

BIRMINGHAM has indeed reason to be proud of its generosity; the Hospital Saturday Fund this year amounted to £9,859, just short of five figures, but still £1,000 more than last year. The sums apportioned were as follow: General Hospital, £2,874 19s. 3d.; Queen's Hospital, £1,524 11s. 8d.; General Dispensary, £1,021 0s. 2d.; Children's Hospital, £787 7s. 8d.; Eye Hospital, £832 4s.; Birmingham and Midland Counties Sanatorium, £345 12s. 3d.; Homœopathic Hospital, £290 13s. 3d.; Women's Hospital, £328 5s. 4d.; Lying-in Charity, £106 16s. 8d.; Orthopædic Hospital, £209 11s.; Ear and Throat Infirmary, £95 2s. 6d.; Dental Hospital, £52 4s. 7d.; Skin and Lock Hospital, £162 15s.; District Nursing Society, £65 6s. 2d.; Jaffray Surburban Branch Hospital, £705 10s. 6d. We tender our hearty congratulations to all the Birmingham charities, whose thanks are due to every worker of the Hospital Saturday Committee.

---

A LARGE concourse assembled at Guy's for the prize-giving to the medical students. The report was read by the dean of the school (Dr. Perry), who said the bright prospects spoken of at the last distribution had been more than realized. There were now 144 students, against 120 last year. There were 42 men living in the college recently opened, but there was accommodation for 58. The competitors in the hospital's examinations had been more numerous than last year. Mr. Hoare then distributed the awards. The treasurer's gold medal for clinical medicine was gained by Mr. A. Ellis Durham (London) and Mr. J. Bryant (Ilminster, *Proximé accessit*); the treasurer's gold medal for clinical surgery, by Mr. F. W. Hall (Sydney); Gurney Hoare prize for clinical study, by Mr. R. D. Mothersole (Colchester); Beaney prize for pathology, by Mr. J. Fawcett (Westgate-on-Sea); Golding Bird prize and medal by Mr. T. G. Stevens (Newington Green); Michael Harris prize for anatomy by Mr. R. H. Ritchie (New South Wales); and the Arthur Durham prizes for dissection by Mr. H. W. Collier, first year's student (Peckham), and by Mr. J. B. Leathes, second year's student (Rochester).

THE Mayor of Maidenhead, Mr. James D. M. Pearce, J.P., offered at a meeting of several gentlemen and leading tradesmen of the town to hand over his Kidwells Park estate to the borough for a recreation and pleasure ground. The park is twelve acres in extent, is situated in the heart of the town, and is worth £1,000 an acre. The munificent offer was accepted, and a trust appointed in accord with the deed of gift.

---

IT is quite useless for the members of the "Seamen's Hospital Society" to talk of their "branch hospital at the Albert Docks." "Baby Dreadnought," the new building, has been named by its friends, and "Baby Dreadnought" it will remain for many years we foresee. Probably in the future it will do as well as its mother, and outgrow the old name and the old home, but for the present we can wish it no better fortune than to fulfil its title; a pet name is ever a sign of a thing beloved.

---

HIS Royal Highness the Prince of Wales, K.G., has graciously accepted the post of President of the Congress of Hygiene and Demography. It is to be hoped his Royal Highness knows where to put the accent on that second word; the meaning of it is kindly explained in the circular: "The aim of the Congress is to awaken public interest in the progress of hygiene and demography, by which latter term is understood the study of the life conditions of communities from a statistical point of view; to afford persons interested in these subjects an opportunity of meeting, with the object of advancing their progress; and, by conferences and debates, to elucidate questions relating to hygiene, demography, and public health." The Congress will be held in London next year.

---

DR. A. BRODIE COCHRANE, who is acting as *locum tenens* at Rotherhithe Infirmary, writes to the Southwark Board of Guardians to complain of his diet, though he professes to be in no way fastidious, having roughed it all over the globe. He says: "I have found the tea and the coffee undrinkable, and have provided myself with supplies of these at my own expense. The butter is rank, and, in my opinion, unfit for food, so I have had to abolish it from my table, and eat dry bread or toast. The milk is very frequently sour—so often that it is a common occurrence. As to the eggs, I have not tasted a new-laid one since I have been here. Though some of those provided I have found passably eatable, many of them have been so stale that I was quite unable to eat them. I had two at breakfast to-day—both stale." There is scarcely a dweller in town, whether in infirmary or in private house, who could not repeat these plaints about eggs and milk.

---

OF course everyone is reading Stanley's book (or extracts from it), and the medical profession will be pleased with the laudation bestowed on Surgeon Parke, even if some of them consider it fulsome. Surgeon Parke's own book is being eagerly watched for by his medical brethren. An amusing part of "In Darkest Africa" is the description of the pigmy damsel who so admired the doctor. "The little thing had performed devoted service to Surgeon Parke, who had quite won her heart with those soft, gentle tones of his that made everybody smile affectionately on the doctor. She used to be the guardian of his tent, and whenever the doctor had to absent himself for his duties she crouched at the door, faithful as a spaniel, and would permit no intruder to approach the doorway. On the road she carried the doctor's satchel, and on nearing the resting place she was as industrious as a bee in collecting fuel, and preparing the surgeon's cheering cup of tea, which after patient teaching she learned was necessary for his well-being."

